# Semaphorin 5B mediates synapse elimination in hippocampal neurons

**DOI:** 10.1186/1749-8104-4-18

**Published:** 2009-05-23

**Authors:** Timothy P O'Connor, Katie Cockburn, Wenyan Wang, Lucia Tapia, Erin Currie, Shernaz X Bamji

**Affiliations:** 1 Department of Cellular & Physiological Sciences & the Brain Research Centre, University of British Columbia, Vancouver, British Columbia, Canada

## Abstract

**Background:**

Semaphorins are known to play an important role in axon guidance and growth by triggering dynamic rearrangements of the actin cytoskeleton in the neuronal growth cone. Intriguingly, some of these guidance molecules are persistently expressed after axonal pathfinding and target recognition are completed. Although their function at these later stages is poorly understood, recent findings suggest a role for these proteins in regulating synaptic connections.

**Results:**

Here we demonstrate that semaphorin 5B (Sema5B) regulates the elimination of synaptic connections in cultured hippocampal neurons. We show that Sema5B is proteolytically processed in neonatal brains and primary hippocampal cultures, resulting in the secretion of Sema5B fragments that include the biologically active semaphorin domain. Overexpression of full-length Sema5B in hippocampal neurons reduces synapse number while expression of a Sema5B construct lacking the semaphorin domain has no effect. Moreover, bath application with the proteolytically processed, secreted fragments containing the semaphorin domain of Sema5B, results in a rapid elimination of synaptic connections as demonstrated by time-lapse imaging. Conversely, depletion of endogenous Sema5B using RNA interference results in a significant increase in synapse number as well as a significant increase in the size of presynaptic and postsynaptic compartments.

**Conclusion:**

Our results demonstrate that in addition to its role as a guidance cue, Sema5B regulates the development and maintenance of synapse size and number in hippocampal neurons. In addition, proteolytic cleavage of Sema5B results in the release of a potentially diffusible semaphorin domain that is a necessary component for its biological function in the regulation of synapse morphology.

## Background

The formation of synaptic connections between growing axons and their appropriate targets is essential for proper functioning of the nervous system. During development, synaptic networks are refined through the elimination of excessive or inappropriate synapses that have initially been formed [1-3]. In addition to these early refinement events, the assembly and disassembly of synapses persists into adulthood [4]. It has been suggested that this continuous modification of synaptic connections may serve as a cellular substrate for learning and memory [5,6] however the molecular mechanisms regulating synapse formation and elimination remain largely unknown.

The semaphorins are a large family of guidance molecules that are involved in processes such as cell migration, axonal guidance, and axonal fasciculation [7]. While the majority of semaphorins act as inhibitory or repellent molecules, some semaphorins also function as permissive or attractive cues [8-10]. Semaphorins typically confer their inhibitory activity through a conserved 500 amino acid 'sema' domain [9], while a variety of alternative functions are associated with their varied C-terminal domains.

Several members of the semaphorin family have recently been shown to affect the structure and function of central nervous system (CNS) synapses. The invertebrate transmembrane protein, Sema1A, is required for the formation of the giant fiber synapse in *Drosophila melanogaster*, but overexpression of this protein results in synaptic destabilization [11]. In mammals, bath application of the secreted semaphorins, Sema3A and Sema3F, decreases and increases, respectively, synaptic transmission in cultured hippocampal neurons [12,13]. Signaling through the Sema3F receptor plexin A3 is also necessary for the elimination of inappropriate synaptic contacts between dentate gyrus mossy fibers and cornu ammonis (CA)3 pyramidal cells during normal hippocampal development [14].

The semaphorin Sema5B is expressed in a variety of regions in the developing brain including the hippocampus [15-17]. Its persistent expression at postnatal stages suggests that Sema5B may contribute to the functioning of neuronal circuits and specifically to the regulation of synaptic functions. Sema5B is a transmembrane protein that, in addition to the typically inhibitory sema domain, possess seven thrombospondin repeats [15,16]. This is intriguing as thrombospondins are permissive for axon outgrowth and enhance synaptogenesis, suggesting a bifunctional role for Sema5B [15,18]. Indeed, the Sema5B homologue, Sema5A, has previously been shown to function as a permissive or inhibitory cue to axon outgrowth depending on local matrix proteoglycans [19]. Whether Sema5B may have similar bifunctional characteristics is unknown.

Here, we provide evidence that Sema5B is involved in synapse elimination in hippocampal neurons. Overexpression of green fluorescent protein (GFP)-Sema5B in hippocampal neurons results in a decrease in synapse number, however this effect is eliminated when the repulsive sema domain is removed. Accordingly, bath application of hippocampal neurons with secreted Sema5B fragments containing the sema domain resulted in the elimination of postsynaptic, postsynaptic density (PSD)-95 clusters. Conversely, depletion of endogenous Sema5B using short hairpin RNA (shRNA) resulted in the exuberant formation and/or maintenance of synaptic connections, with a concomitant increase in the size of pre and postsynaptic densities. These data therefore reveal a new role for semaphorins in regulating synapse maintenance and morphology and provide insights into the role that these guidance molecules may play in the developing brain.

## Results

### Sema5B antibody

**Figure 1:**
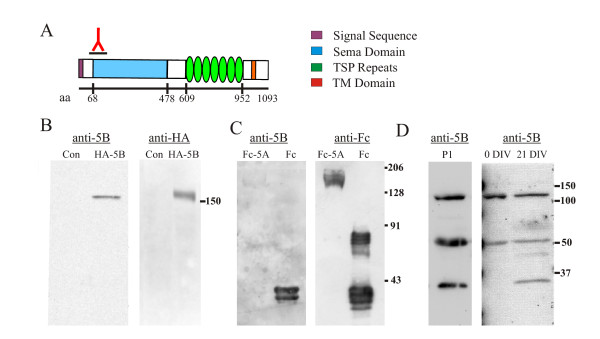
**Sema5B antibody specificity**. **(a) **Schematic illustration of Sema5B including the region in the N-terminus used to generate the antibody. The amino acids bordering the sema domain and thrombospondin repeats are indicated. **(b) **Lysates from human embryonic kidney (HEK)293 cells transfected with hemaglutinin (HA)-Sema5B were separated by SDS-PAGE and immunoblots probed with antibodies specific to Sema5B or HA. Antibodies recognize a predominant band at approximately 150 kDa corresponding to HA-Sema5B. **(c) **Anti-5B does not recognize a full-length mouse Sema5A extracellular domain Fc-human fusion protein (Sema5A-Fc). Purified Sema5A-Fc and Fc proteins (kindly provided by Dr D Sretavan) were separated by SDS-PAGE and immunoblots probed with antibodies specific to Sema5B (anti-5B) or anti-human Fc chain (anti-Fc). Some crossreactivity was observed in the Fc lane, most likely between the anti-rabbit IgG secondary and the large amount of human Fc loaded on the gel. Sema5A-Fc ran at the expected molecular weight of ~170 Kda. **(d) **Sema5B is proteolytically processed *in vivo*. Lysates from P1 mouse brains and hippocampal neurons cultured for 0 and 21 days *in vitro *(DIV) were separated by SDS-PAGE and immunoblots probed with anti-5B. Numerous repeatable bands were observed indicating proteolytic processing of Sema5B in the nervous system. N = 4 brains.

**Figure 2:**
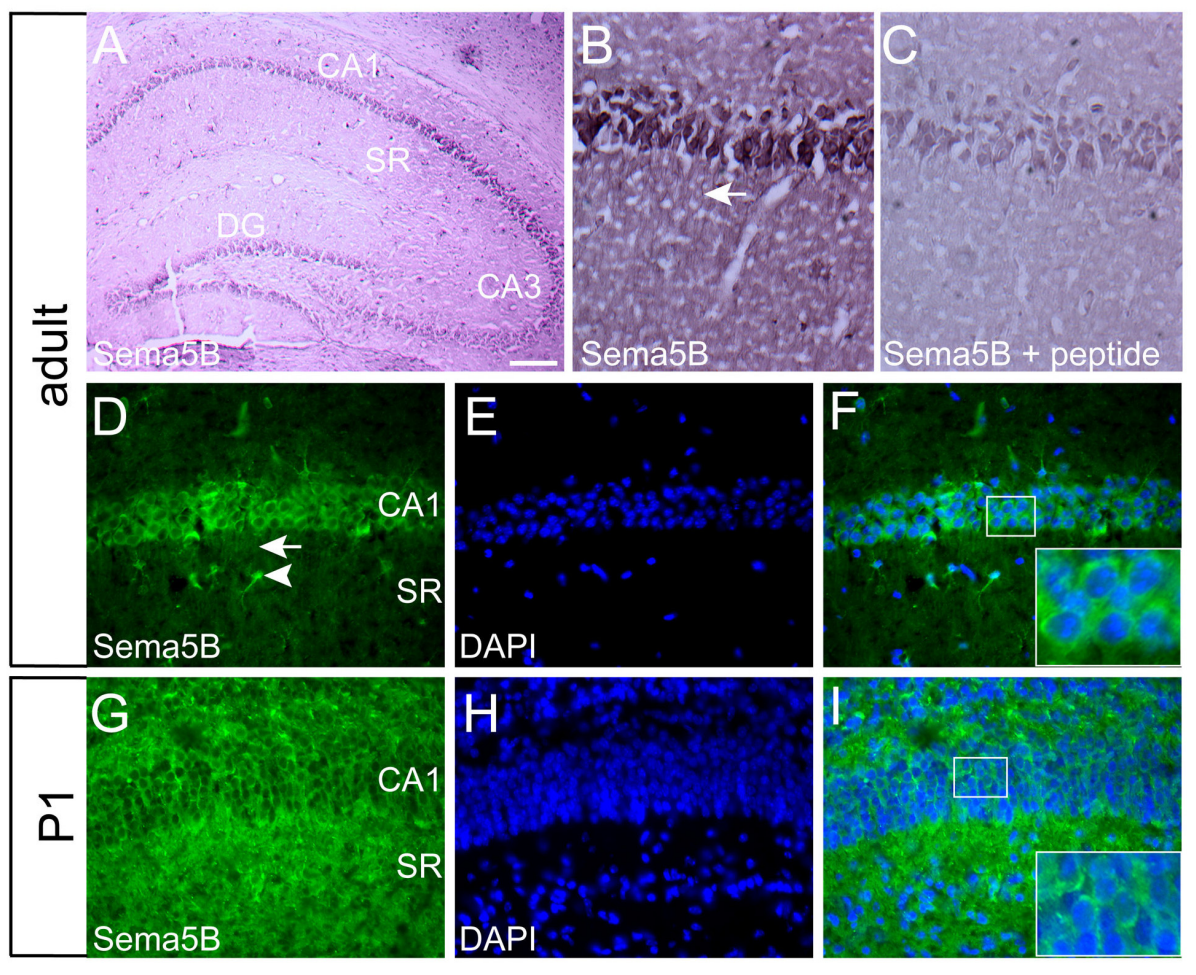
**Sema5B is expressed in the hippocampus**. **(a-i) **Immunolabeling of coronal brain sections demonstrating Sema5B expression in cornu ammonis (CA)1-CA3 hippocampal pyramidal neurons and in the dentate gyrus. Preadsorption with the peptide used in the generation of the Sema5B antibody resulted in reduced immunocytochemical labeling, confirming the specificity of this antibody (b, c). Sema5B was localized along the length of the neurites ((b, d), arrows) and in a subset of cells in the stratum radiatum ((d), arrowhead). (a-i) N = 4 brains. Scale bar; (a) 200 μm, (b-i) 40 μm.

Antibodies were generated against the N-terminus of full length Sema5B (anti-5B) along a region that showed the least amino acid homology with other semaphorins including Sema5A (Figure 1a). This antibody recognized a hemaglutinin (HA)-tagged recombinant form of Sema5B at approximately 150 kDa (Figure 1b) but did not crossreact with a purified Sema5A (Figure 1c). In contrast, a 150 kDa Sema5B band was absent in postnatal day 1 (P1) cortical or hippocampal lysates, but several smaller bands were apparent indicating that the majority of Sema5B is proteolytically processed (Figure 1d). Similar bands were observed in 21 days *in vitro *(DIV) and freshly cultured hippocampal neurons (Figure 1d). The anti-5B antibody recognized a number of bands, all 110 KDa and smaller, indicating that the sema domain is proteolytically cleaved from the full length Sema5B and could potentially act as a diffusible molecule. As further validation of the specificity of the anti-5B antibody, the N-terminal peptide fragment was able to compete with the endogenous Sema5B epitopes (see Figure 2, [Additional file 1]), and there was a reduction of Sema5B immunolabeling in Sema5B knock down cells [Additional file 2].

### Sema5B is expressed in the developing and adult hippocampus

Previous *in situ *hybridization data has shown robust Sema5B mRNA expression in the developing rodent brain that is also maintained in the postnatal hippocampus [15,17]. To confirm this expression, postnatal day 1 (P1) and adult brain sections were immunolabeled for Sema5B (Figure 2a–i). Sema5B was robustly expressed in the CA1-CA3 pyramidal cell layer as well as the dentate gyrus in both neonatal and adult mice (Figure 2a–i). Sema5B was also localized to the stratum radiatum region immediately adjacent to the pyramidal cell layer. This reflected Sema5B localization in the neurites of CA1-CA3 cells, as well as Sema5B expression in interneurons (Figure 2b, d, g). Immunostaining in the stratum radiatum region appeared more robust in P1 mice compared to adults. This may reflect the dynamic nature of synapse formation and elimination at earlier versus later stages. Preadsorption with the peptide used in the generation of the Sema5B antibody resulted in reduced immunocytochemical labeling confirming the specificity of this antibody (Figure 2b, c; [Additional file 1]).

**Figure 3:**
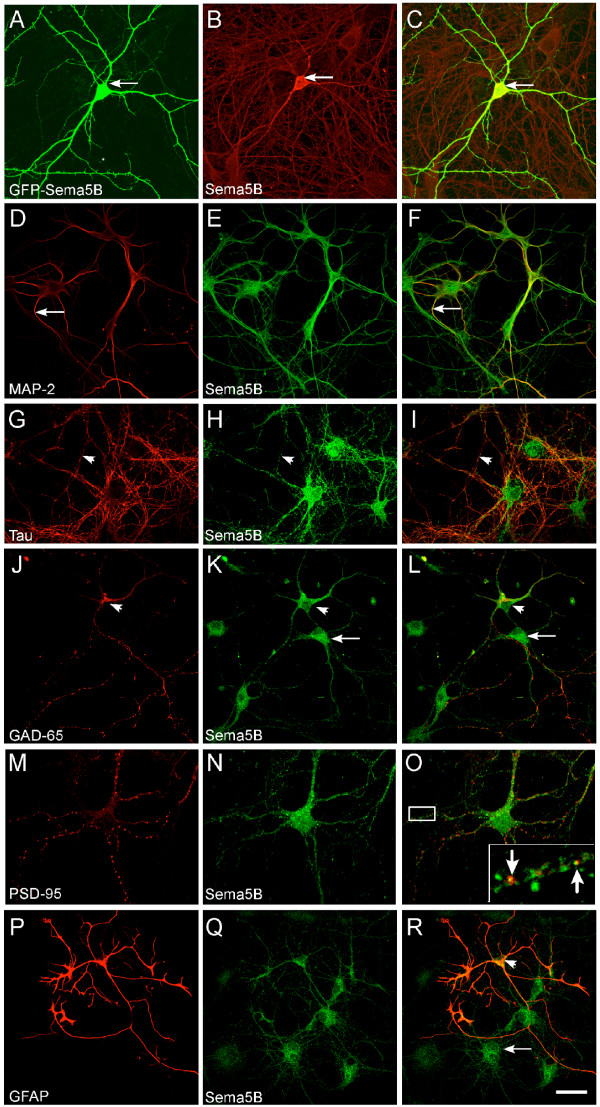
**Sema5B expression in hippocampal neurons**. **(a-r) **Confocal images of 7 to 10 days *in vitro *(DIV) hippocampal cultures immunolabeled with anti-5B antibody plus the specified marker. (a-c) Immunolabeling of a hippocampal neuron transfected with green fluorescent protein (GFP)-Sema5B demonstrated that Sema5B levels were markedly higher in the GFP-Sema5B-expresing cells (arrow) compared to the surrounding, untransfected neurons. (d-i) In untransfected cells, Sema5B was expressed in dendrites labeled with microtubule-associated protein 2 (MAP-2) (arrows, (d-f)) and axons labeled with tau (arrowheads; g-i). (j-l) Both glutamate decarboxylase (GAD)-65-positive (arrowhead) and GAD-65-negative (arrow) cells expressed Sema5B. (m-o) Coimmunolabeling with the postsynaptic marker, postsynaptic density (PSD)-95, demonstrated minimal colocalization (insert, arrows), demonstrating that Sema5B is not particularly enriched at synapses. (p-r) Sema5B expression was also observed in glial fibrillary acidic protein (GFAP)-positive glial cells. Scale bar; a-l 40 μm, m-o 20 μm and p-r 40 μm.

The specificity of the 5B antibody was also demonstrated by immunolabeling hippocampal neurons transfected with full-length Sema5B fused to GFP (GFP-Sema5B). Indeed, Sema5B levels were markedly higher in GFP-Sema5B-expresing cells compared to surrounding, untransfected neurons, which expressed endogenous Sema5B (Figure 3a–c). In untransfected cells, Sema5B exhibited a punctate distribution in the cell body and colocalized with both MAP-2 (Figure 3d–f) and tau (Figure 3g–i) indicating its localization in both dendrites and axons, respectively. Immunolabeling with the inhibitory marker glutamate decarboxylase (GAD)-65 revealed that both GAD-65-positive and GAD-65-negative cells express Sema5B. This indicates that Sema5B is expressed by both inhibitory and excitatory cells (Figure 3j–l). As the punctate distribution along the neurites was suggestive of a synaptic distribution, cells were also labeled with antibodies against the excitatory postsynaptic marker, PSD-95 (Figure 3m–o) and the presynaptic marker, synaptophysin (data not shown). There did not appear to be an enrichment of Sema5B at synaptic compartments although there was some minimal colocalization observed between Sema5B and PSD-95 (Figure 3o, inset). Finally, Sema5B expression was also observed in glial fibrillary acidic protein (GFAP)-positive glial cells (Figure 3p–q).

### Overexpression of GFP-Sema5B decreases synapse number

**Figure 4:**
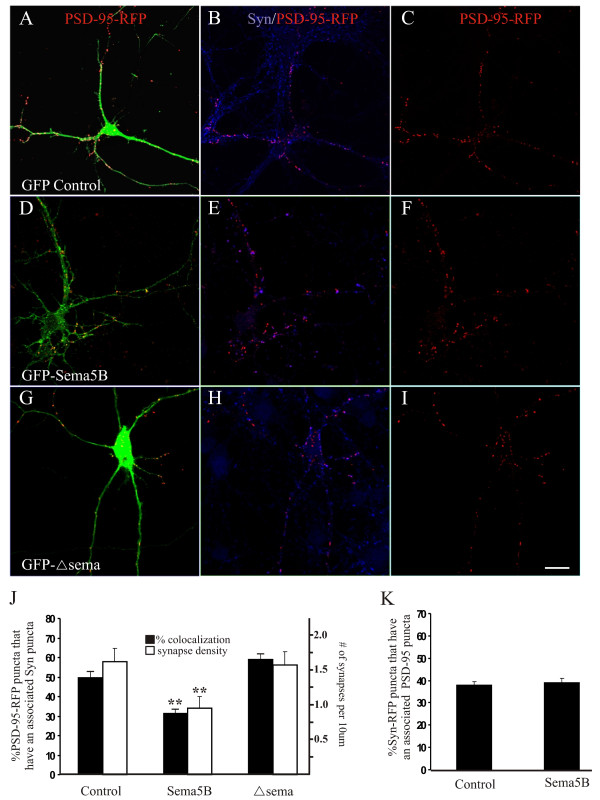
**Overexpression of Sema5B reduces the number of synaptic inputs**. **(a-i) **Confocal images of 13 days *in vitro *(DIV) hippocampal cultures cotransfected at 10 DIV with postsynaptic density (PSD)-95-RFP plus green fluorescent protein (GFP) (a), GFP-Sema5B (d), or GFP-Δsema (g), and immunolabeled for synaptophysin (b, e, h). Although the number of PSD-95-red fluorescent protein (RFP) puncta remained constant (c, f, i), there was a significant decrease in the number of synaptic inputs onto Sema5B overexpressing cells as measured by a decrease in the percentage of PSD-95 puncta that had an associated synaptophysin puncta (j; Student's t test; *P *< 0.005). Similarly there was a correlative decrease in the density of synaptic inputs (j; Student's t test; *P *< 0.05). The number and density of synaptic inputs onto cells overexpressing Sema5BΔsema was not significantly different than control (j; Student's t test; *P *> 0.5). The number of synapses being formed by Sema5B overexpressing cells, as measured by the number of PSD-95 puncta that colocalized with Syn-RFP (data not shown), was unchanged (k; Student's t test; *P *> 0.5). N = at least 15 neurons per condition from 3 separate cultures. Scale bar 20 μm.

**Figure 5:**
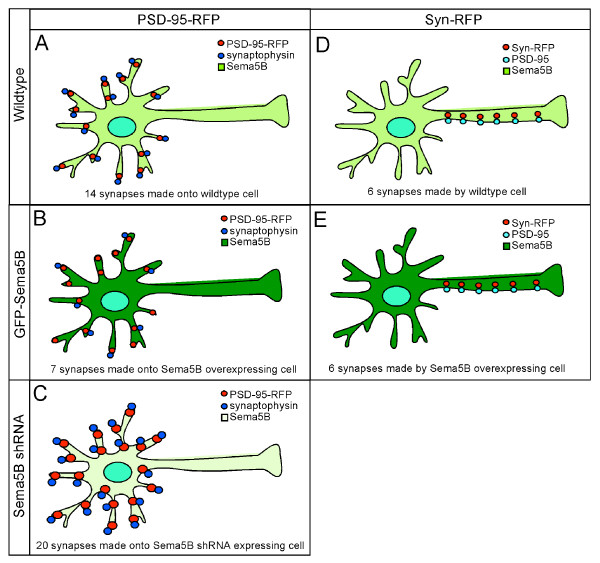
**Schematic illustration of Sema5B function at synapses**. Cells expressing high Sema5B levels exhibit fewer presynaptic inputs **(b) **compared to wild type cells **(a)**. Consistent with these observations, a reduction in Sema5B levels results in a significant increase in the number of presynaptic inputs and the size of both pre and postsynaptic compartments **(c)**. In contrast, the number of synapses made by cells expressing high levels of Sema5B **(e) **is similar to wild type cells **(d)**.

To investigate the function of Sema5B at synapses, GFP-Sema5B was expressed in cultured hippocampal neurons (Figure 4). Immunolabeling with GFP antibodies of non-permeabilized human embryonic kidney (HEK)293 cells expressing GFP-Sema5B showed that it was appropriately targeted to the cell membrane (data not shown). To examine the effects of GFP-Sema5B overexpression on synapses, neurons were cotransfected with the excitatory postsynaptic marker PSD-95 fused to red fluorescent protein (RFP) to visualize postsynaptic densities. Cultures were then fixed and immunolabeled for synaptophysin, and synapses identified by the colocalization of the pre and postsynaptic markers synaptophysin and PSD-95-RFP, respectively (Figure 4b, e, h). Although the number of PSD-95-RFP puncta were similar between control and Sema5B overexpressing cells (311.5 ± 30.4; 266.1 ± 35.84, respectively, *P *= 0.33), the percentage of PSD-95 puncta that have an associated synaptophysin puncta, and the density of PSD-95/synaptophysin puncta was significantly lower in GFP-Sema5B expressing cells compared to control cells (Figure 4j). This indicated that the number of synaptic inputs from wild type neurons onto the Sema5B overexpressing cell was significantly attenuated (see model, Figure 5).

To determine the number of synapses being made by the Sema5B overexpressing cells onto wild type neurons, cells were cotransfected with GFP-Sema5B and the presynaptic marker synaptophysin-RFP and then immunolabeled for PSD-95. The number of synaptophysin puncta in control and Sema5B-expressing cells was similar (201.2 ± 37.4; 180.1 ± 18.1, respectively) and no significant difference was observed in the percentage of synaptophysin puncta that have an associated PSD-95 puncta (Figure 4k). Therefore, although overexpressed Sema5B is distributed to both axons and dendrites, it is the increased expression in dendrites that results in a reduction in synapse number (see model, Figure 5).

### The sema domain of Sema5B is required for the decrease in synapse number

The sema domain of semaphorins typically acts to mediate the biological actions of these guidance molecules [9]. To determine whether this domain plays a role in regulation of synapse elimination, hippocampal neurons were transfected with Sema5B lacking the sema domain (GFP-5BΔsema). Despite a robust expression of GFP-5BΔsema throughout the neuron, we observed no effect on the density and proportion of PSD-95-RFP puncta that have an associated synaptophysin puncta (Figure 4). This indicates the semaphorin domain of Sema5B is largely responsible for the reduction in synapse number following Sema5B overexpression.

### Treatment of hippocampal neurons with supernatant from HEK293 expressing Sema5B results in synapse elimination

#### Sema5B stimulates growth cone avoidance and collapse of young hippocampal neurons

**Figure 6:**
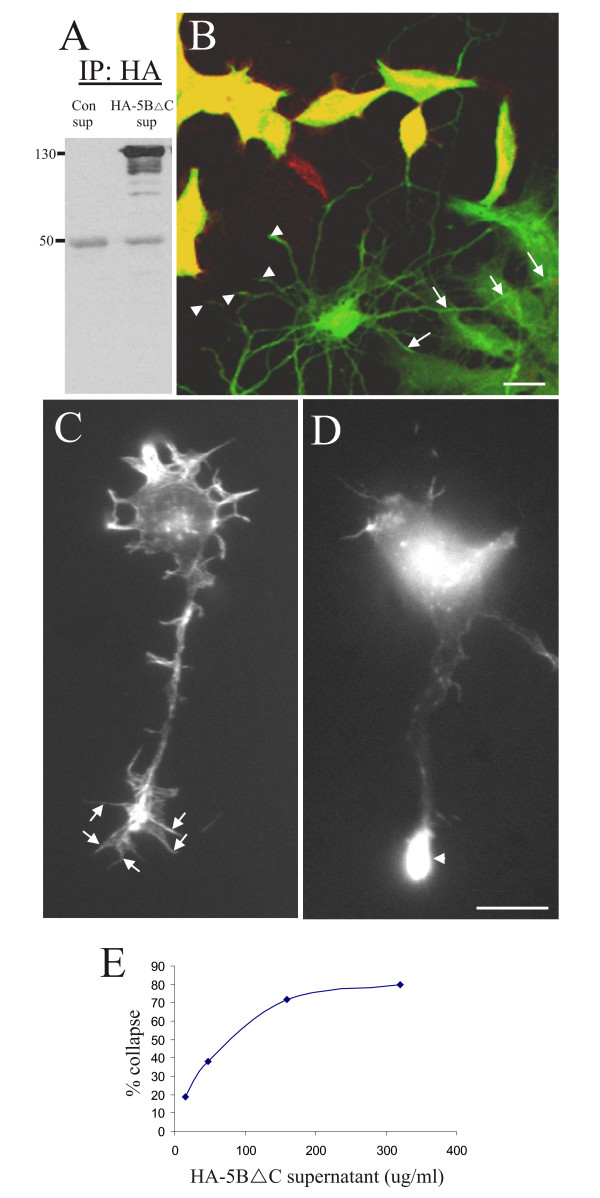
**Hippocampal neurons respond to secreted Sema5B**. **(a) **Supernatant from human embryonic kidney (HEK)293 cells overexpressing hemaglutinin (HA)-Sema5BΔC was immunoprecipitated using an HA antibody, separated by SDS-PAGE, and immunoblots probed with anti-HA antibody. N = 4. **(b) **Hippocampal neurons (2 days *in vitro *(DIV)) were cocultured with HEK293 cells transfected with HA-Sema5BΔC and immunolabeled with anti-HA (red) and anti-neurofilament (green) antibodies (anti-neurofilament antibodies also label HEK293 cells). Collapsed hippocampal growth cones (arrowheads) avoid HEK293 cells overexpressing HA-Sema5BΔC (yellow). In contrast, neurites extend along non-transfected HEK293 cells (arrows). **(c, d) **Hippocampal neurons (1 DIV) treated with supernatant from HEK293 cells expressing HA-Sema5BΔC exhibit collapsed growth cones ((d), arrowhead) compared to cells treated with untransfected HEK293 cell supernatant, which had typical growth cones that extended numerous filopodia ((c), arrows). N > 6 assays. **(e) **An example of a single collapse bioactivity assay. Collapse activity was determined at least twice for each aliquot of Sema5B-containing supernatant. Scale bar 20 μm.

Numerous semaphorins and their receptors are proteolytically processed, and in the case of Sema4D, the sema domain is cleaved from the full-length protein resulting in a secreted peptide [20-22]. Our data suggests that similar processing of Sema5B occurs in the hippocampus (Figure 1d). To further verify that hippocampal neurons can respond to Sema5B, neurons were cocultured with HEK293 cells transiently transfected with HA-Sema5B or a variety of truncated/deletion mutants. After 2 days in culture hippocampal neurites were observed to avoid contact with HEK293 cells expressing constructs that contained the sema domain (Figure 6b, arrowheads), but grew robustly over control, untransfected HEK293 cells (Figure 6b, arrows). While the majority of neurites avoid HEK293 cells expressing Sema5B, on occasion one or two neurites will contact and cross transfected cells (Figure 6b; see also [17]). Nonetheless, the high degree of avoidance observed suggested that Sema5B is biologically active for hippocampal neurons. The most robust repulsion was observed when hippocampal neurons were cocultured with HEK293 cells that expressed a form of Sema5B that had the entire intracellular C-terminus removed (HA-Sema5BΔC) (Figure 6b). Although the avoidance of Sema5BΔC-expressing cells by hippocampal neurites could be due to contact mediated repulsion, the collapse of growth cones several microns away from the cells could be due to a short diffusible signal (Figure 6b). To test this possibility, hippocampal neurons were bathed with concentrated supernatant from HEK293 cells expressing HA-Sema5BΔC. Bath application resulted in the collapse of neurites in a dose-dependent manner (Figure 6d, e), whereas supernatant from untransfected cells had no impact on growth cone collapse (Figure 6c). These observations indicate that hippocampal neurons are responsive to Sema5B and exhibit a collapsed/avoidance response similar to that observed with dorsal root ganglion neurons [23]. In addition, these data indicate that the collapse and/or repulsion of processes are due to a biologically active fragment of Sema5B that is released into the media from the transfected HEK293 cells. To confirm the presence of a secreted form of Sema5B, the supernatant from cells expressing HA-Sema5BΔC was concentrated, run on an SDS-PAGE gel, and probed with an anti-HA antibody. A number of bands were observed between 100 and 130 kDa with one prominent band at approximately 130 kDa (Figure 6a). These N-terminal fragments are sufficiently large enough to contain the entire semaphorin domain of Sema5B. We were unable to concentrate soluble secreted fragments from hippocampal culture supernatants despite our observations that hippocampal tissue lysates showed the presence of these fragments (Figure 1d). This may reflect the low concentrations of secreted sema fragments in hippocampal cultures.

#### Bath application with Sema5B results in synapse elimination in older hippocampal cultures

**Figure 7:**
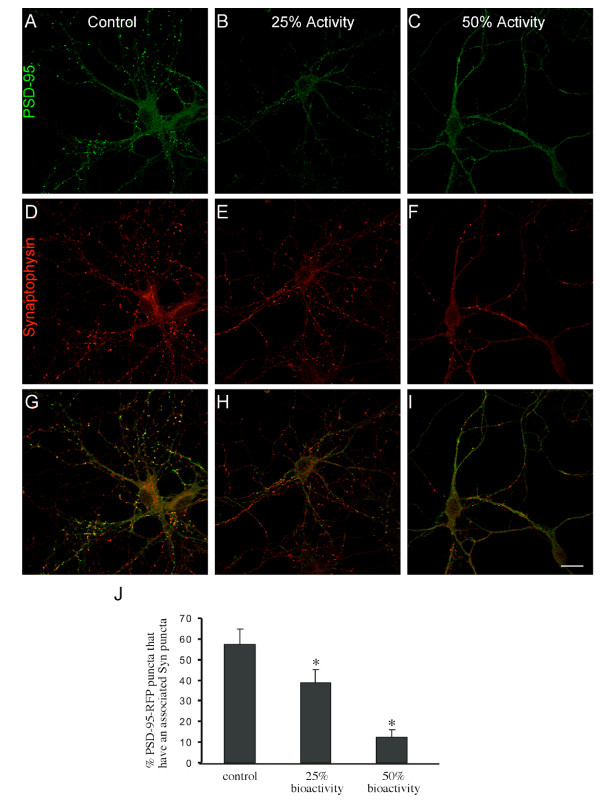
**There is a decrease in synapse number in cells treated with secreted Sema5B**. **(a-i) **Confocal images of 9 to 10 days *in vitro *(DIV) hippocampal neurons treated with supernatant from hemaglutinin (HA)-Sema5BΔC-expressing human embryonic kidney (HEK)293 cells and immunolabeled with anti-postsynaptic density (PSD)-95 (a-c) and anti-synaptophysin (d-f) antibodies. Effective concentrations were determined by growth cone collapse assays on 1 DIV hippocampal neurons, and reflect concentrations that confer 25% or 50% of maximum growth cone collapse. (g-j) Synapse number was determined by the number of synaptophysin puncta that colocalized with PSD-95. N = 25 neurons from 4 separate cultures. Scale bar 20 μm.

**Figure 8:**
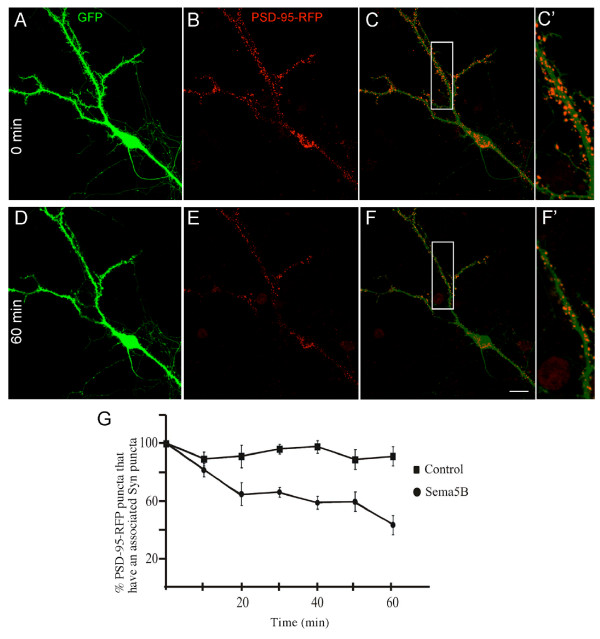
**Postsynaptic density (PSD)-95 puncta are eliminated upon treatment with secreted Sema5B**. **(a-f) **Representative time-lapse confocal images of a 9 to 10 days *in vitro *(DIV) hippocampal neuron transfected with green fluorescent protein (GFP) (a, d) and PSD-95-red fluorescent protein (RFP) (b, e) and treated with secreted Sema5B (50% bioactivity). (c, f) Merged images of (a), (b) and (d), (e), respectively. There was a significant reduction of the number of PSD-95-RFP puncta while little change was observed in neurite morphology (compare (c') with (f')). **(g) **Bath application of secreted Sema5B for 1 h resulted in an elimination of approximately 50% of PSD-95-RFP puncta. N = 4 cells per condition. Scale bar 25 μm.

Based on the observation that overexpression of GFP-Sema5B, but not GFP-5BΔsema, results in the reduction in synapse number, we investigated whether the secreted sema domain of Sema5B has an effect on the generation and/or stability of synapses. To determine the effect of supernatant treatment on synaptogenesis, 9 to 10 DIV hippocampal cultures were treated with supernatant from 293 cells expressing HA-Sema5BΔC (Figure 7). Specifically, a quarter to a half of the concentration of supernatant that produced maximal growth cone collapse in the collapse assay was used for bath application. After bath application, a reduction in the number of synapses, identified by the colocalization of synaptophysin and PSD-95 puncta, was observed (Figure 7g–j). Increasing the supernatant decreased the percentage of PSD-95 puncta that had an associated Syn puncta twofold, from approximately 40% to 10% after 2 h (Figure 7c, f, i, j). This effect did not appear to be secondary to collapse, as there were few examples of neurite collapse observed despite the loss of synaptic sites (see Figure 8). To confirm these observations, experiments were repeated using time-lapse imaging. Neurons expressing GFP to visualize neurite morphology and PSD-95-RFP to visualize postsynaptic densities were treated with the supernatant of HA-Sema5BΔC-transfected HEK293 cells and imaged every 10 minutes for 60 to 90 minutes following bath application. At 1 h after treatment there was a 56% decrease in the number of PSD-95-RFP puncta (Figure 8). In control cells, only minimal fluctuations in the number of PSD-95 puncta were observed over time (Figure 8g). These fluctuations may be attributed to biological changes in protein localization over time, or slight drifts in focal planes. Bleaching of fluorophores is unlikely as exposure times were short, and laser intensity minimal. The reduction of PSD-95 puncta in response to HA-Sema5BΔC bath application was not secondary to decreased cell viability, as no difference in survival was observed using the dye YO-PRO-1 as an early apoptotic marker, or using the highly sensitive colorimetric 3-(4,5-dimethylthiazol-2-yl)-2,5-diphenyltetrazolium bromide (MTT) cell viability assay (data not shown).

### Sema5B protein knockdown increases synapse number and size

**Figure 9:**
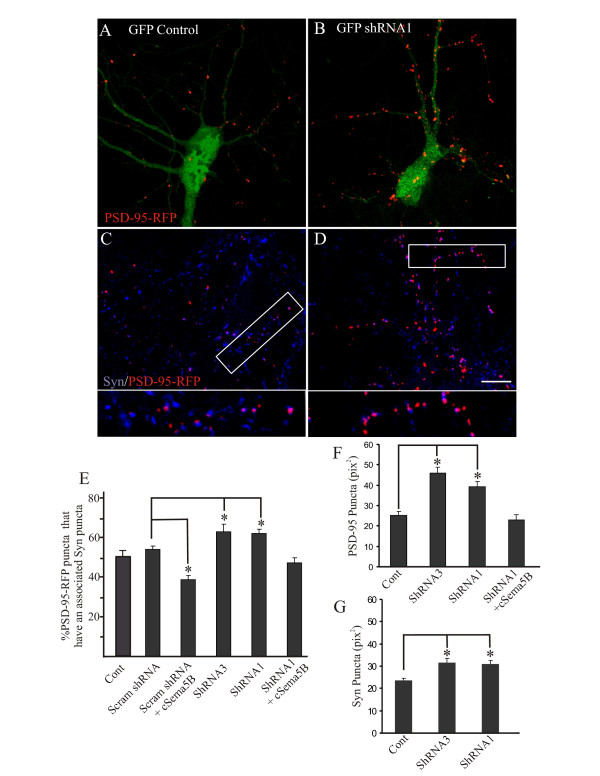
**Sema5B knockdown increases synapse number and size**. **(a-d) **Confocal images of 9 to 10 days *in vitro *(DIV) hippocampal neurons expressing postsynaptic density (PSD)-95-red fluorescent protein (RFP) plus either green fluorescent protein (GFP) (a) or GFP-short hairpin RNA (shRNA) (b). Cells were immunolabeled with anti-synaptophysin and synapse number determined by the percentage of PSD-95-RFP puncta that had an associated synaptophysin cluster (c-e). Similar to our previous observations, coexpression of a chick Sema5B with a scrambled shRNA resulted in a significant decrease in synapse number (compare **(e) **to Figure 4j; Student's t test, *P *< 0.001). In contrast, knockdown of Sema5B resulted in a significant increase in synapse number ((e); Student's t test shRNA1 and 3, *P *< 0.005). The increase in synapse number with Sema5B shRNA was abrogated by coexpression with an shRNA-insensitive chick Sema5B (e). In addition to an increase in synapse number, there was a twofold increase in the size of PSD-95-RFP puncta in Sema5B knockdown cells (Student's t test shRNA1 and 3, *P *< 0.001) that was rescued by coexpression of a chick Sema5B (**(f)**; Student's t test, *P *= 0.462 compared to control). A similar increase in the size of synaptophysin puncta associated with Sema5B knockdown was observed (**(g)**; Student's t test shRNA1 and 3, *P *< 0.005). N = at least 25 cells per condition from at least 3 separate cultures. Scale bar 10 μm.

To further examine the role of Sema5B at the synapse, Sema5B protein levels were attenuated using shRNAs. One of the known caveats of shRNA is the 'off-target' effects, where unintended transcripts are silenced [24]. To minimize this possibility, two different shRNAs were used to reduce Sema5B protein levels. Previous reports have demonstrated a significant knockdown of Sema5B by Sema5B shRNA1 and Sema5B shRNA3 in heterologous cells and brain tissue [17]. We confirmed Sema5B knockdown in primary hippocampal neurons using immunocytochemistry (see [Additional file 2]). To determine the effect of Sema5B knockdown on synapse number, neurons were cotransfected with PSD-95-RFP and cultures immunolabeled for synaptophysin (Figure 9). Expression of both shRNA constructs resulted in a modest yet significant increase in the number of synapses as identified by PSD-95/synaptophysin colocalization (Figure 9b, d, e), while the number of synapses in cells expressing control scrambled shRNA were similar to that of wild type neurons (Figure 9a, c, e). In addition, the size of PSD-95-RFP puncta in Sema5B knockdown neurons (Figure 9c, d, f) and synaptophysin puncta apposing Sema5B knockdown neurons (Figure 9c, d, g) was significantly increased. Increased clustering of PSD-95 has been shown to drive synapse maturation by recruiting a number of PDZ domain-containing proteins to postsynaptic sites, which in turn results in an increased accumulation of synaptophysin presynaptically and an increase in miniature excitatory postsynaptic currents (mEPSCs) (PDZ is an acronym combining the first letters of three proteins first discovered to share the domain: PSD-95, *Drosophila *disc large tumor suppressor (DlgA), and zonula occludens-1 protein (zo-1))[25].

To further address the possibility that the observed increase in synapse number and size may result from off-target effects of the shRNAs, we determined whether this phenotype could be rescued by overexpressing an shRNA-resistant chick isoform of Sema5B in shRNA-expressing neurons. Overexpression of chick Sema5B completely abolished the shRNA-mediated increase in synapse number and size (Figure 9e, f).

## Discussion

Although individual synapses can be stable for prolonged periods of time, synaptic connections also retain a remarkable capacity to be rapidly disassembled and reformed. The formation and elimination of synaptic connections are particularly prominent during early development, but continue in the adult, and are thought to be essential for the cellular processes underlying learning and memory [1,26]. Considerable efforts have focused on the mechanisms underlying synaptogenesis; however, relatively little is known about the molecular mechanisms that control the elimination of synaptic contacts. The speed at which synapse disassembly occurs has been shown to be more rapid than the normal rates of protein turnover at the synapse, indicating that synapse disassembly is not only caused by the lack of maintenance of synapses, but rather by mechanisms that actively drive synapse breakdown. For example, the half-life of α-amino-3-hydroxyl-5-methyl-4-isoxazole-propionate (AMPA) receptors at a synapse has been measured to be 18 to 23 h [27] and yet live-imaging studies have demonstrated that synapses containing AMPA receptors can be eliminated in as quickly as 90 minutes [28].

Our data demonstrates an important role for Sema5B in the elimination of synaptic connections. Overexpression of full-length, wild type Sema5B in hippocampal neurons resulted in a significant reduction in the number of synapses. Surprisingly, the functional regulation appears to be limited to the sema domain as synapse number remained unaffected following overexpression of Sema5B lacking this domain. Using time-lapse imaging of hippocampal neurons bathed in media containing secreted Sema5B fragments, we determined that the decrease in the number of synaptic inputs is not due to failure to form synapses, but rather in a rapid elimination of synapses. Together with the observation that knockdown of Sema5B in cells increases the number and size of synapses, these data demonstrate that Sema5B is expressed and secreted by hippocampal neurons to regulate presynaptic input.

### Proteolytic processing of Sema5B

A number of Sema5B N-terminal fragments between 110 and 30 kDa were detected by western blot analysis of cortical and hippocampal tissue. Based on our observations of Sema5B expression in other tissues and in heterologous cell lines, full length Sema5B is approximately 150 kDa. This would suggest that in the cortex and hippocampus, Sema5B is cleaved extracellular to the transmembrane domain, releasing an N-terminal protein that most often contains the entire sema domain. This type of cleavage has been observed in another transmembrane semaphorin, Sema4D [22]. Sema4D can be cleaved at the plasma membrane and released into the extracellular environment, resulting in both a membrane-bound and a secreted molecule. A similar cleavage event may regulate Sema5B release in hippocampal neurons, allowing the sema domain to exert its effect in both a paracrine and autocrine manner. Whether Sema5B can function over longer distances is unknown. Many semaphorins, including Sema5A and B, exhibit complex interactions with the extracellular matrix [19,29,30]. In particular, Sema5A and B have been shown to bind to heparin and chondroitin sulfate proteoglycans (CSPG) and in the case of Sema5A, this binding regulates its function [19]. Similarly, the punctate distribution of secreted semaphorins along the cell surface and their interactions with their receptor neuoropilin-1 appears to be regulated by proteoglycan binding [29]. Whether similar interactions with the extracellular matrix plays a role in localizing Sema5B to hippocampal synapses is unknown.

Presently, little is known about the Sema5B receptor. While plexin B3 has been shown to function as a receptor for Sema5A in heterologous cells [31], an alternative, unknown receptor appears to function in neurons [19]. Identifying the Sema5 receptor(s) is complicated by their interactions with proteoglycans. For example, the binding affinity of Sema5B to CSPG and heparin can be regulated by the level and location of sulfation on the glycosaminoglycan side chain, suggesting an additional level of complexity in regulating Sema5B function [30]. Indeed, Sema5A has been shown to have complete opposite functions depending on whether it is bound by CSPG or heparin [19]. This suggests the importance of the extracellular matrix (ECM) molecules in potentially regulating Sema5B interactions with its receptor.

### Endogenous localization of Sema5B

While other semaphorins have been shown to effect synapse function in vertebrate neurons [12,13,32,33], the absence of specific antibodies has not allowed for an examination of their distribution. Nonetheless, other guidance molecules, such as the ephrins and their receptors and repulsive guidance molecules (RGM), have been shown to colocalize and function at the synapse [34-37]. Here we demonstrate that at least the N-terminal fragments of Sema5B are localized predominantly in the cell body and along dendrites and axons, with modest accumulation observed at synaptic sites. It is possible that Sema5B is proteolytically processed within the cell and the sema domain-containing fragment secreted or that Sema5B is cleaved by extracellular proteases following membrane insertion of the secretory vesicle. It would be of interest to determine how this proteolytic process is regulated and whether this process is modulated in active versus inactive synapses as well as in mature versus developing circuits. It has yet to be definitively demonstrated whether Sema5B is being released by presynaptic or postsynaptic cells; however, our data suggest that Sema5B is being released postsynaptically to regulate presynaptic inputs (discussed below). Our observations suggest that it is the local release of the N-terminal fragment that is important for Sema5B regulation of synapse integrity, however we cannot rule out a function for the C-terminal fragment. Indeed, thrombospondins have previously been shown to play a role in enhancing synaptogenesis [18] and the C-terminal fragment of Sema5B consists of a number of thrombospondin repeats. Nonetheless, we could find no effect on synapse number or size when we overexpressed a C-terminal fragment that was lacking the sema domain.

### The role of Sema5B in synapse disassembly

Overexpression of GFP-Sema5B resulted in a significant decrease in the number of synaptic inputs being formed on these cells. This was determined by cotransfecting cells with PSD-95-RFP to identify postsynaptic compartments in Sema5B-overexpressing cells and then immunostaining for the presynaptic marker, synaptophysin. Interestingly, when cells were cotransfected with Sema5B and synaptophysin-RFP to label presynaptic compartments, and immunolabeled with PSD-95 to identify synapses, no change in synapse number was observed. Therefore, our data support the conclusion that Sema5B fragments are being secreted from postsynaptic cells to affect their presynaptic partners. Indeed, although the number of PSD-95-RFP puncta is similar in control and Sema5B overexpressing cells, the proportion of synaptophysin associated with these puncta is reduced. It is unlikely that Sema5B is being secreted presynaptically as the number of synapses being formed by the Sema5B overexpressing neuron onto wild type cells remains constant. This is in accordance with data from the cerebellum and the mammalian neuromuscular junction (NMJ) demonstrating that elimination is induced by the postsynaptic cell, and that presynaptic disassembly precedes postsynaptic disassembly [1,38]. Indeed, it has been proposed that in hippocampal neurons, Sema3A is released from postsynaptic sites and binds to its receptor neuropilin 1 (NP-1) localized to the presynaptic membrane, resulting in a significant reduction in synapses. Although it was reported that extracellular signal-regulated kinase (ERK) phosphorylation occurred in response to Sema3A, the signaling pathway that resulted in synapse disassembly was not identified [12].

If the cell is secreting increased Sema5B fragments, why doesn't it impact the number of its synaptic outputs? Two explanations are possible. First, it is possible that Sema5B fragments do not diffuse freely, but remain local by its association with ECM molecules to mediate its effects locally. This would afford a higher degree of spatial resolution for synapse elimination. Second, it is possible that a sufficient concentration of freely diffusing Sema5B is not reaching synapses being formed by axons and their targets.

Overexpression of GFP-Sema5B resulted in a loss of presynaptic markers apposed to postsynaptic densities that remain similar in number. In contrast, treatment of neurons with supernatant from HEK293 cells resulted in the rapid loss of postsynaptic markers. This discrepancy is most likely due to the concentration of Sema5B fragments bathing the cell. Moreover, this may reflect the temporal nature of presynaptic and postsynaptic disassembly.

### Sema5B signaling and synapse elimination

The actin cytoskeleton is known to play a key role in the development and maintenance of young synapses. Presynaptically, treatment of neurons with latrunculin A results in a reduction in the number and size of synaptophysin-labeled clusters [39]. At the postsynaptic compartment, actin depolymerization has been reported to elicit the internalization [40] and diffusion [41] of neurotransmitter receptors away from the synapse. Moreover, actin regulates the morphology and plasticity of dendritic spines, the postsynaptic compartment for the majority of excitatory synapses. Interestingly, long-term depression induces a shift in the spine F-actin/G-actin ratio to G-actin, and a concomitant decrease in spine head volume with the occasional disappearance of some spines [42]. The actin cytoskeleton may therefore be a principal target for stabilizing or destabilizing signals that ultimately result in synapse maintenance or elimination.

Although little is known about the receptor(s) involved in transducing Sema5B function, Sema5B has recently been shown to stimulate growth cone collapse by inducing the disassembly of adhesion complexes and the actin meshwork in growth cones [23]. This disassembly has been shown to be due to the concurrent activation of calpain and calcineurin [23], and it is possible that similar pathways may be activated during synapse disassembly. The protease calpain is particularly intriguing as its activity appears to function in regulating synaptic efficacy and structure [43-45]. Moreover, a number of synaptic structural and signaling proteins are known substrates for calpain, including PSD-95, β-catenin and the actin nucleating proteins cortactin and Wiskott-Aldrich syndrome protein (WASP) [43,46-49], suggesting a direct possible mechanism for Sema5B regulation of synapse structure. Whether similar pathways observed during growth cone collapse are stimulated during synapse remodeling have not been tested.

## Materials and methods

### Antibody generation

Antibodies were generated against the N-terminus (amino acids 20 to 131) of Sema5B. Homology comparisons showed the least amount of conservation between semaphorins along this peptide length. Recombinant peptide was produced using the glutathione-S-transferase (GST) fusion system (Amersham Pharmacia Biotech, Baie d'Urfe, QC, Canada)) and was purified with a glutathione agarose affinity column (Sigma, Oakville, ON, Canada). Female New Zealand white rabbits were immunized for antibody generation. For the first injection, 0.5 ml of 1 mg/ml recombinant peptide was mixed with 0.5 ml Freund's complete adjuvant. For boosters, 0.1 mg of recombinant peptide in 0.5 ml was mixed with 0.5 ml of incomplete Freund's adjuvant. Adjuvant and protein were emulsified before injection. Booster injections were given every 2 weeks and antibody generation was monitored with test bleeds and western blot analysis against the fusion peptide. Typically, terminal bleeds were collected after three boosters. The polyclonal antibodies were immunoaffinity purified with AminoLink columns (Fisher, Nepean, ON, Canada).

### Recombinant DNA

Full-length Sema5B was inserted into pEGFP-C1 (Clonetech, Mountain View, CA, USA) at the *Hin*dIII and *Sac*II sites. Sema5BΔsema (lacking amino acids 1 to 551) was generated using standard PCR and similarly subcloned into pEGFP-C1. Full-length chick Sema5B and Sema5BΔC (lacking amino acids 1,001 to 1,093) were subcloned into the *Xma*I/*Sac*II sites of the HA epitope tagged pDisplay expression vector (Invitrogen, Burlington ON, Canada) to express an N-terminal tagged HA fusion protein. Multiple stop codons were positioned before the platelet derived growth factor receptor (PDGFR) transmembrane region of the vector. The PSD-95-RFP and synaptophysin-RFP constructs were kind gifts from D Bredt (Eli Lilly & Co., Indianapolis, IN, USA) and L Reichardt (Dept. Physiology, UCSF, CA, USA), respectively. Generation of shRNA vectors was performed as described previously [17]. The following sequences were targeted: shRNA1 (561) = 5'-GGACTATTGAGAAGATC AA and shRNA3 (1,664) = 5'-GAAGACAGTT CCAACATGA. One targeted sequence generated an incorrect final insert sequence with no homology to Sema5B and was therefore used as a scrambled control. Oligoduplex palindromes were cloned into the *Xho*I/*Hpa*I restriction sites of pLentilox 3.7, which contains an internal ribosomal entry site (IRES) GFP sequence downstream of the cloning site. Resultant shRNA vectors were tested for their ability to knock down Sema5B expression by immunocytochemistry of transfected hippocampal cells.

### Neuronal cultures

Rat hippocampi from E18 fetal rats were prepared as previously described [50] and plated at a density of 130 cells/mm^2^. Neurons were transfected using lipofectamine 2000 at 7 to 8 DIV as per the manufacturer's recommendation. All transfected cells were fixed and analyzed 2 to 3 days after transfection. For HEK cell/hippocampal neuron cocultures, wild type HEK cells or cells expressing GFP-Sema5B were plated at approximately 50 cells/mm^2^. At 24 h later, freshly dissociated hippocampal neurons were overlaid onto dishes at a density of 130 cells/mm^2^.

### Immunohistochemistry

#### Brain sections

Adult male and P1 mice (P30-P60) were anesthetized and perfused with phosphate-buffered saline (PBS) (pH 7.4), followed by 4% paraformaldehyde (PFA) in PBS. The brains were dissected out and post-fixed in 4% PFA for 2 h before going through a series of sucrose-PBS solutions (10% to 30%). Whole brains were embedded in TissueTek (Sakura Finetek, Torrance, CA, USA) and frozen in liquid nitrogen. The tissue was cut into 12 μm thick coronal sections using an HM 500 cryostat (Microm Instruments, San Marcos, CA, USA). Vectastain immunocytochemistry was performed as described previously [51] Briefly, sections were permeabilized in 0.1% Triton-X for 30 minutes and blocked in 4% goat serum for 20 minutes before incubation with anti-5B overnight at 4°C. For preadsorption experiments, Sema5B N-terminal peptide (amino acids 20 to 131) was added in excess (1 to 3 times the concentration of anti-5B antibody) to an aliquot of anti-5B and allowed to mix for 1 h before applying to tissue. After overnight incubation a sections were washed and then incubated with goat anti-rabbit biotin-conjugated secondary antibody (1:200, Vector Laboratories, Burlingame, CA, USA) for 1 h at room temperature. Labeling was visualized using the Vectastain ABC peroxidase and Vector VIP kits (Vector Laboratories) as per the manufacturer's recommendation. For fluorescent immunocytochemistry sections were incubated with a polyclonal goat anti-rabbit Alexa Fluor 488-conjugated secondary antibody (1:500; Molecular Probes, Eugene, OR, USA). Sections were then dehydrated and mounted in Permount (Fisher).

#### Hippocampal neuron cultures

Neuron cultures were fixed in 4% paraformaldehyde/4% sucrose for 10 minutes, permeabilized in 0.1% Triton-X for 10 minutes, and then blocked in 10% goat serum for 1 hr at room temperature. Primary antibodies were applied in 1% goat serum overnight at 4°C and secondary antibodies were applied in 1% goat serum for 1 hr at room temperature. Primary antibodies were mouse anti-synaptophysin (Sigma) and mouse anti-PSD-95 (ABR, Golden, Co, USA). Secondary antibodies were Alexa Fluor 488, Alexa Fluor 633, and Texas Red-conjugated goat anti-mouse or goat anti-rabbit antibodies (Molecular Probes).

#### Sema5B bath application

For collapse assays, supernatant was collected from wild type HEK293 cells, or cells expressing HA-Sema5B, GFP-Sema5B, or a recombinant Sema5B that had a C-terminal deletion of 100 amino acids (HA-Sema5BΔC). For synapse elimination assays, supernatant was collected from wild type HEK293 cells or cells expressing HA-Sema5BΔC. The advantage of using the HA-Sema5BΔC was that a significantly greater amount of proteolytically cleaved Sema5B (containing the sema domain) was released into the media. Supernatants were concentrated 100 times by Centricon concentrators (Millipore, Billerica, MA, USA) with 30 kDa cut-offs. Bioactivity of the concentrated supernatant was determined by measuring growth cone collapse 1 h after addition to 1 DIV hippocampal neurons as described previously [52]. Briefly, growth cones lacking lamellipodia and extending four or less filopodia were considered collapsed. For each batch of supernatant, experiments were performed twice with at least 20 randomly selected growth cones scored per experiment. Concentrated supernatant that stimulated 50% and 25% growth cone collapse was used for bath application of hippocampal cultures.

#### Image analysis and quantification

Transfected hippocampal neurons and fluorescent immunocytochemical brain sections were imaged using an Olympus Fluoview 1000 confocal microscope (60 ×/1.4 Oil Plan-Apochromat; Olympus, Markham, ON, Canada). All images in a given experiment were captured with the same exposure time and conditions. Brightfield vectastain immunocytochemical images were captured using an Axioplan 2 Imaging microscope (Zeiss, Jena, Germany) equipped with a Retiga 1350X camera (Quantitative Imaging Corporation, Burnaby, British Columbia, Canada) and Northern Eclipse software (Empix Imaging, Mississauga, Ontario, Canada).

#### Quantification of puncta colocalization

Images of cells immunolabeled for Sema5B, synaptophysin, or PSD-95 were analyzed using ImageJ with a colocalization plug-in downloaded from the program's website [53]. Following thresholding, points of colocalization were defined as regions greater than 4 pixels in size where the intensity ratio of the two channels was greater than 50%. Numbers of colocalized puncta were expressed as a percentage of the total number of puncta, quantified using the same threshold and size criteria and excluding colocalized points that appeared to be within the cell body. The average numbers of colocalized synaptic puncta in each treatment group were analyzed for statistical significance using the Student's t test.

#### Quantification of PSD-95-GFP puncta following Sema5B bath treatment

Neurons expressing PSD-95-GFP were bathed in supernatant from wild type HEK293 cells, or cells expressing HA-Sema5BΔC. For each experiment the concentrated supernatant was diluted in 100 μl culture media just prior to addition to the cells. Cells were imaged every 10 minutes for 60 to 90 minutes immediately following sema bath application. Images were imported into Image J where they were thresholded and analyzed.

#### Immunoblot analysis

Hippocampal and cortical tissues, as well as cultured hippocampal neurons were lysed in approximately 4 volumes (w/v) of buffer containing (50 mM Tris pH 7.4, 150 mM NaCl, 1.0% NP-40, 10% glycerol). Extracts were run using standard SDS-PAGE, and immunoblots probed with anti-Sema5B. Proteins were visualized using enhanced chemiluminence on a Bio-Rad Versadoc 4000.

## Competing interests

The authors declare that they have no competing interests.

## Authors' contributions

TPOC and SXB conceived the study, evaluated the findings, prepared the figures and manuscript. KC, WW, LT and EC carried out all the experimental procedures.

## Supplementary Material

Additional file 1 **Sema5B is expressed in the adult hippocampus**. Coronal section of an adult brain immunolabeled with anti-5B antibody **(a)**, or immunolabeled with anti-5B antibody after peptide preadsorption **(b)**. Click here for file

Additional file 2 **Sema5B short hairpin RNA (shRNA) decreases Sema5B expression in hippocampal neurites**. **(a-c) **Confocal image of a 10 days *in vitro *(DIV) hippocampal neuron transfected with green fluorescent protein (GFP)-Sema5B shRNA at 8 DIV (a), and immunolabeled with anti-5B antibody (b). Images of anti-5B antibody labeling were overexposed to ensure adequate detection of Sema5B, resulting in an apparent loss of punctate distribution. Both transfected ((a, c), arrows) and untransfected ((b, c), arrowheads) neurites are observed. Isolated transfected neurites (arrows) display a clear reduction in Sema5B immunoreactivity compared to untransfected neurites (arrowheads). Asterisks indicate close apposition of transfected and untransfected neurites (a, c). Scale bar 20 μm. Click here for file
