# Supplementary figures and images for: Semaphorin 5B mediates synapse elimination in hippocampal neurons
Source: Neural Dev. 2009 May 23;4:18. doi: 10.1186/1749-8104-4-18 (PMC2696441; doi:10.1186/1749-8104-4-18)

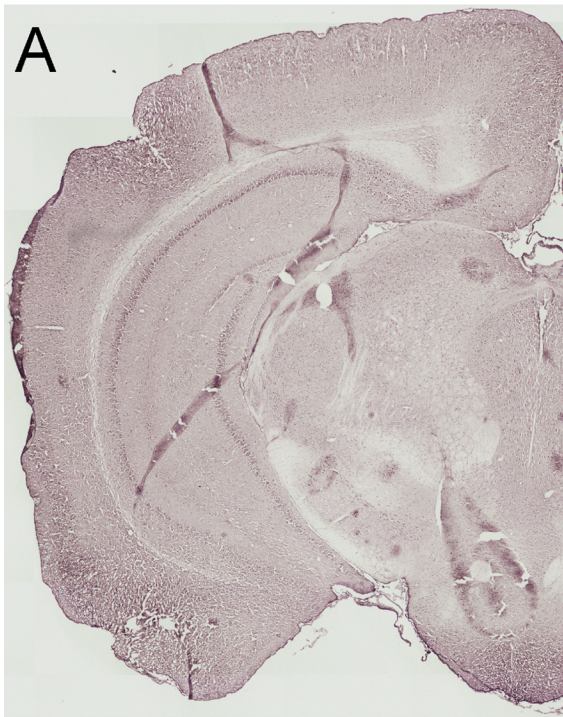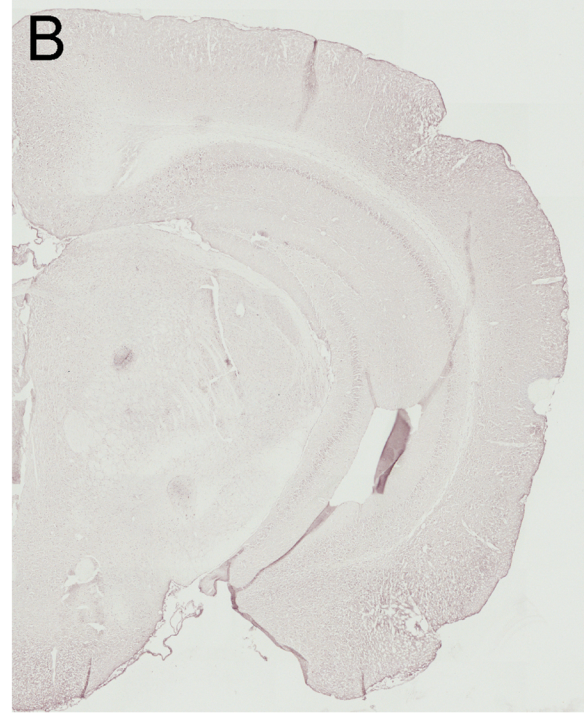

Additional Figure 1

Supplement: Additional file 1 — Sema5B is expressed in the adult hippocampus. Coronal section of an adult brain immunolabeled with anti-5B antibody (a), or immunolabeled with anti-5B antibody after peptide preadsorption (b). [file 1749-8104-4-18-S1.pdf]

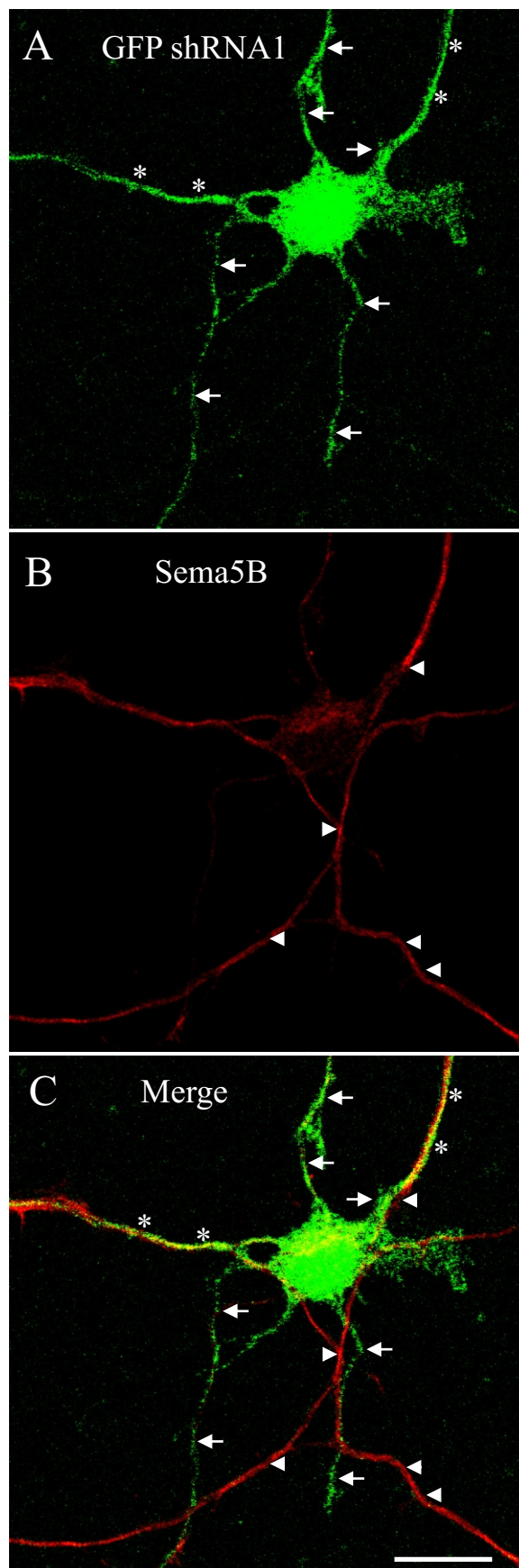

Additional Figure 2

Supplement: Additional file 2 — Sema5B short hairpin RNA (shRNA) decreases Sema5B expression in hippocampal neurites. (a-c) Confocal image of a 10 days in vitro (DIV) hippocampal neuron transfected with green fluorescent protein (GFP)-Sema5B shRNA at 8 DIV (a), and immunolabeled with anti-5B antibody (b). Images of anti-5B antibody labeling were overexposed to ensure adequate detection of Sema5B, resulting in an apparent loss of punctate distribution. Both transfected ((a, c), arrows) and untransfected ((b, c), arrowheads) neurites are observed. Isolated transfected neurites (arrows) display a clear reduction in Sema5B immunoreactivity compared to untransfected neurites (arrowheads). Asterisks indicate close apposition of transfected and untransfected neurites (a, c). Scale bar 20 μm. [file 1749-8104-4-18-S2.pdf]
